# Of mice and men: molecular genetics of congenital heart disease

**DOI:** 10.1007/s00018-013-1430-1

**Published:** 2013-08-10

**Authors:** Troels Askhøj Andersen, Karin de Linde Lind Troelsen, Lars Allan Larsen

**Affiliations:** Wilhelm Johannsen Centre for Functional Genome Research, Department of Cellular and Molecular Medicine, University of Copenhagen, Blegdamsvej 3, 2200 Copenhagen, Denmark

**Keywords:** Congenital heart disease, CHD, Disease genes, Copy number variants, CNVs

## Abstract

Congenital heart disease (CHD) affects nearly 1 % of the population. It is a complex disease, which may be caused by multiple genetic and environmental factors. Studies in human genetics have led to the identification of more than 50 human genes, involved in isolated CHD or genetic syndromes, where CHD is part of the phenotype. Furthermore, mapping of genomic copy number variants and exome sequencing of CHD patients have led to the identification of a large number of candidate disease genes. Experiments in animal models, particularly in mice, have been used to verify human disease genes and to gain further insight into the molecular pathology behind CHD. The picture emerging from these studies suggest that genetic lesions associated with CHD affect a broad range of cellular signaling components, from ligands and receptors, across down-stream effector molecules to transcription factors and co-factors, including chromatin modifiers.

## Introduction

Congenital heart disease (CHD) is the most prevalent birth defect, with a postnatal incidence of 0.8 % [1] and an approximately tenfold higher prenatal incidence [2]. CHD is a group of structural abnormalities of the heart, which include septal defects, valve defects and lesions affecting the outflow tract. The treatment of CHD has improved during the last 50 years, and today 95 % of CHD patients survive to adulthood, which has resulted in a growing population of adults living with CHD [3].

The etiology of CHD is complex and is associated with both environmental and genetic causes. Genetically, CHD is a very heterogeneous disease; 55 human disease genes have been identified so far (Table 1 and text below), however, experiments with targeted deletions in mice have revealed more than 500 genes which lead to heart defects when mutated (http://www.informatics.jax.org/). Thus it is likely that at least the same number of human CHD disease genes exist.

**Table 1:** Genes associated with CHD via intragenic mutations

HCNG gene symbol (alternative symbol)	Protein function^a^	Type of CHD^b^	Reference
Genes encoding transription factors			
*CITED2*	Transcriptional co-activator	I	[18]
*FOXH1*	Forkhead box TF	I	[29]
*FOXP1*	Forkhead box TF	I	[246]
*GATA4*	GATA-binding TF	I	[89–91]
*GATA6*	GATA-binding TF	I	[247, 248]
*IRX4*	Iroquois homeobox TF	I	[249]
*MED13L*	Multiprotein coactivator subunit	I	[250]
*NKX2*-*5*	Homeobox TF	I	[69–71]
*NKX2*-*6*	Homeobox TF	I	[251]
*TBX1*	T-box TF	S (DiGeorge syndrome)	[156]
*TBX5*	T-box TF	S (Holt–Oram syndrome)	[102, 103]
*TBX20*	T-box TF	I	[104]
*SALL4*	Zinc finger TF	I, S (Duane-radial ray syndrome)	[252–255]
*TFAP2B*	AP-2 TF	I, S (Char syndrome)	[110–112]
*ZFPM2*	Zinc finger TF	I	[255, 256]
*ZIC3*	Zinc finger TF	HTX	[19]
Genes involved in cell signaling			
*ACVR1*	Activin receptor, type 1	I	[257]
*ACVR2B*	Activin receptor 2B	HTX	[14]
*BRAF*	Serine/threonine protein kinase	S (NS, LS, CFC)	[56, 64]
*CBL*	E3 ubiquitin ligase	S (NS-like)	[66]
*CFC1*	Ligand (EGF family)	HTX	[17]
*GDF1*	Ligand (BMP/TGFbeta family)	HTX	[16]
*HRAS*	RAS GTPase	S (Costello syndrome)	[63]
*JAG1*	NOTCH ligand	S (Alagille syndrome)	[37, 38]
*LEFTY2* (*LEFTYA*)	Ligand (BMP/TGFbeta family)	HTX	[14]
*KRAS*	RAS GTPase	S (NS, CFC)	[56, 57]
*MAP2K1* (*MEK1*)	MAP kinase kinase	S (CFC)	[64]
*MAP2K2* (*MEK2*)	MAP kinase kinase	S (CFC)	[64]
*NF1*	Negative regulator of RAS-MAPK signalling	S (neurofibromatosis-NS)	[67]
*NRAS*	RAS GTPase	S (NS)	[62]
*NODAL*	Ligand (BMP/TGFbeta family)	HTX	[12, 13]
*NOTCH1*	NOTCH receptor	I	[45–47]
*NOTCH2*	NOTCH receptor	S (Alagille syndrome)	[41, 42]
*PTGFRA*	PTGFRα receptor	I	[258]
*PTPN11*	Protein tyrosine phosphatase	S (NS)	[55]
*RAF1*	MAP kinase kinase kinase	S (NS, LS)	[60, 61]
*RIT1*	Ras-related GTPase	S (NS)	[259]
*SHOC2*	RAS-MAPK modulator	S (NS)	[65]
*SMAD6*	BMP/TGFbeta modulator	I	[260]
*SOS1*	Guanine nucleotide exchange factor (RAS-MAPK pathway)	S (NS)	[58, 59]
*TAB2*	Activator of MAP3K7 (TAK1)	I	[236]
*TDGF1*	Co-receptor for TGF-β ligands	I	[29]
Genes encoding structural proteins			
*ACTC1*	Cardiac α-actinin	I	[124]
*ELN*	Elastin	I^c^	[184]
*MYH6*	Cardiac myosin HC	I	[118–120]
*MYH7*	Cardiac myosin HC	I	[123]
*MYH11*	Smooth muscle myosin HC	I	[125]
Genes encoding epigenetic regulators			
*CHD7*	Binding to H3K4Me3	S (CHARGE syndrome)	[134, 135, 137]
*KMT2D* (*MLL2*)	H3K4 methyltransferase	S (Kabuki syndrome)	[132]
*EP300*	Histone acetyltransferase	S (Rubinstein–Taybi syndrome)	[117]
*CREBBP*	Histone acetyltransferase	S (Rubinstein–Taybi syndrome)	[116]
*EHMT1*	H3K9 methyltransferase	S (Kleefstra syndrome)	[205, 208]
Other genes			
*CRELD1*	Cell adhesion	I	[261]
*MCTP2* ^*d*^	Possible role in Ca^2+^ signalling	I	[262]
*NPHP4*	Ciliary protein	I, HTX	[20]

The list include genes, which have been associated with CHD by identification of mutations in two or more unrelated patient and/or genes where human genetic analyses are complemented with functional analyses

^a^ *TF* transcription factor, *HC* heavy chain

^b^ *I* isolated CHD, *S* syndromic CHD, *HTX* heterotaxy, *NS* Noonan syndrome, *LS* LEOPARD syndrome, *CFC* Cardiofaciocutaneous syndrome

^c^Genomic deletions, which include *ELN* cause Williams–Beuren syndrome

^d^Intragenic genomic duplication causing premature truncation at p.F697X

During the last two decades, linkage analysis has been used to successfully identify CHD disease genes in large families, segregating isolated CHD and genetic syndromes, where CHD is part of the phenotypic spectrum (syndromic CHD). Furthermore, fine-mapping of genomic copy number variants (CNVs) in patients with isolated CHD, or CHD in combination with additional birth defects, have been used to identify candidate disease genes. Follow-up studies of candidate genes in animal models, particularly in mice, have been very successful in validating the candidates, and to gain insight into the function of the gene products in heart development.

Identifying disease genes in CHD is critically important to understand the disease. Identification of a novel disease gene or a causative pathway will enhance current knowledge of the molecular biology involved in human cardiac development, and the molecular pathology underlying CHD. Such knowledge may lead to new preventive strategies and perhaps new treatments. Furthermore, such knowledge may also increase our understanding of the factors involved in cardiomyogenic stem cell differentiation, and may thus aid in the development of regenerative therapy for treatment of myocardial infarction. In familial cases of CHD, identification of disease genes will benefit the genetic counseling process for CHD-afflicted families. Such knowledge is particularly important for the growing population of adults with CHD, due to the high recurrence risk of certain forms of CHD [4].

In this review, we aim to summarize current knowledge in the molecular genetics of CHD, from the perspective of, disease gene identification efforts in humans, and functional analyses of disease genes in animal models.

## Part I: genes associated with congenital heart disease

Below we discuss genes associated with syndromic and isolated CHD, juxtaposing studies from multiple model systems to clarify why errors in the underlying molecular machinery manifests themselves as congenital heart defects.

### Genes associated with laterality defects

The heart is the first organ to break the bilateral symmetry of the developing embryo. During early embryogenesis, left–right asymmetry of the body-axis is established via intricate cross-talk amongst signaling pathways such as Notch, Nodal, Hedgehog, FGF and BMP, ultimately restricting NODAL signaling to the left side of the embryo. The nodal cilia model is the predominant model to elucidate induction of embryonic asymmetry in the developing embryo, although other models have been proposed [5]. Briefly, nodal cilia in the node of the primitive streak produce a directional fluid flow which induces left–right asymmetry by delivering morphogens to the left side of the embryo and/or by acting on mechanosensory cilia [6–9]. Ultimately, laterality cues are relayed downstream to ensure left-sided expression of the PITX2 transcription factor in the lateral plate mesoderm [10], a critical component in determining organ laterality [11]. *NODAL* [12, 13], *LEFTY2* [14], *ACVR2B* [15], *GDF1* [16] *CFC1* [17], *CITED2* [18] and *ZIC3* [19] have all been localized to the laterality signaling pathway. Albeit human mutations in these genes show a wide range of heart malfunctions, many cluster around laterality defects such as heterotaxy and faulty looping of the heart. Interestingly, a recent study identified mutations in *Nephronophthisis*-*4* (*NPHP4*), a cilia related gene, and linked them to a variety of cardiac laterality defects such as transposition of the great arteries (TGA), atrioventricular septal defects (AVSD), double outlet right ventricle (DORV), dextrocardia and abnormal pulmonary venous return. Laterality defects of the abdominal organs were also observed. Morpholino knock down of *nphp4* in zebrafish resulted in reverse orientation or faulty looping of the heart [20]. Previous studies have also connected other NPHP family members to inborn heart defects and laterality deficiencies [21, 22].

The *ZIC3* gene encodes a zinc finger transcription factor known to cause cardiovascular defects when mutated in humans. Mutations in *ZIC3* cause X-linked familial heterotaxy but are also found in sporadic cases of heterotaxy and isolated CHDs [19, 23, 24]. Null and heterozygous *Zic3* mice display a variety of cardiac defects such as TGA, interrupted aortic arch (IAA), atrial septum defect (ASD) and ventricular septal defect (VSD) in combination with various other developmental anomalies, thus resulting in a phenocopy of the clinical spectrum of malfunctions found in humans with heterotaxy [25]. How mutations in *ZIC3* result in faulty heart looping is currently unknown. However, recent reports place ZIC3 upstream in the Nodal signal cascade [25, 26] with conditional loss-of-function studies showing that ZIC3 is required in the migrating mesoderm but not for heart progenitors and in the heart compartment [27].

Recently, the transcription factor FOXH1 was outlined as a possible signaling intersection between BMP and Nodal signaling to establish left/right asymmetry [28]. Mutations in *FOXH1* have been linked to human heart defects [29], as well as the lack of outflow tract and right ventricle is seen in *Foxh1* ^−/−^ mouse embryos [30].

In humans, mutations in the transcriptional co-activator CITED2 (Cbp/p300-interacting transactivator, with Glu/Asp-rich carboxy-terminal domain 2) are associated with laterality defects and cardiac anomalies such as septal defects and TGA [18]. Mice deficient in *Cited2* die during gestation expressing partially penetrant laterality defects and fully penetrant heart defects [31, 32]. The heart anomalies include ASD, VSD, common atrioventricular canal (CAVC), DORV and IAA type B [32, 33]. Recently, Lopes et al. [31] showed that specific deletions of *Cited2* in heart progenitors do not produce heart defects and that the cardiac malfunctions seen in *Cited2* ^−/−^ embryos arise during the early phases of establishing the left–right body axis in close relation to NODAL signaling.

### Genes encoding components of signaling pathways

Animal models have illustrated that cardiac development involves spatial and temporal coordination of a number of signaling pathways [34, 35]. The identification of disease genes in syndromic and isolated CHD has confirmed the involvement of a subgroup of these pathways in human heart development, and has further contributed new information about additional pathways.

The NOTCH signaling pathway acts locally as a cell-fate regulator, and as a patterning signal effector in many developmental processes. Its activity includes left–right axis partitioning and heart morphogenesis. For an extensive review on Notch signaling in cardiac development see [36].

Identification of mutations in *JAG1*, encoding a NOTCH signaling ligand, in patients with Alagille syndrome provided the first link between NOTCH signaling and human CHD [37, 38]. Alagille syndrome (AGS, OMIM #118450) is a multisystem disorder, which involves the liver, heart, eyes, face and skeleton [39]. Approximately 90 % of these patients have cardiovascular anomalies, often presenting in the form of stenosis in the pulmonary artery branch, valvular pulmonary stenosis (PS) and tetralogy of Fallot (TOF) [40]. The majority (>90 %) of AGS cases are caused by mutations in *JAG1*, however select (<1 %) cases are caused by mutation in *NOTCH2* [41, 42]. Mice homozygous for targeted deletion of *Jag1* die during embryonic development possibly due to vascular defects while heterozygous *Jag1* ^−*/*+^ mice display ocular defects [43].

In contrast, doubly heterozygous *Jag1* ^+*/*−^, *Notch2* ^+*/*−^ mice exhibit multiorgan abnormalities characteristic of AGS, supporting a genetic interaction between *JAG1* and *NOTCH2* in AGS [44].

Mutations in *NOTCH1* have been identified in patients with isolated CHD [45–47]. Patients with *NOTCH1* mutations often present malfunctions of the aortic valve. NOTCH1 signaling has been linked to endothelial-to-mesenchymal transformation (EMT), a fundamental process in the early stages of cardiac valve formation, where endocardial cells detach to become a migratory mesenchyme that forms endocardial cushions, precursors of cardiac valves. *Notch1* mutant mice develop hypoplastic endocardial cushions due to impaired EMT [48]. Recently, Luna-Zurita et al. and others [48–50] outlined that a NOTCH1, WNT4 and BMP2 signal interplay between the endocardium and myocardium underlie valve morphogenesis. Furthermore, Bosse et al. [51] showed that compound mutant *Notch* ^+*/*−^; *Nos3* ^−*/*−^ mice display an accelerated bicuspid aortic valve phenotype compared to *Notch* ^+*/*−^ and *Nos3* ^−*/*−^ alone, suggesting an interaction between nitric oxide (NO) and NOTCH signaling in the development of the aortic valve. In the same paper, these data were further supported by in vitro data, suggesting that NO regulates Notch signaling in aortic valve interstitial cells.

Signal transduction through the RAS-mitogen activated protein kinase (MAPK) pathway can stimulate cell proliferation, differentiation, survival and metabolism. Identification of disease genes in Noonan syndrome (OMIM #163950), Costello syndrome (OMIM #218040), LEOPARD syndrome (OMIM #151100), Cardio-facio-cutaneous (CFC) syndrome (OMIM #115150) and a few other syndromes with distinct but overlapping phenotypes, collectively known as RASopathies (see [52] for review), have firmly established a link between the RAS-MAPK signal transduction pathway and human CHD. The RASopathies are manifested by a wide range of multisystem anomalies, including CHD. In Noonan syndrome approximately 85 % of patients have a variety of cardiac defects, most commonly including pulmonary valve stenosis, ASD and hypertrophic cardiomyopathy [53, 54].

Linkage analysis and mutation screening has identified mutations in *PTPN11* as the cause of approximately half of cases with Noonan syndrome [55]. Subsequently, mutations in *KRAS* [56, 57], *SOS1* [58, 59], *RAF1* [60, 61], *NRAS* [62], *HRAS* [63], *BRAF* [56, 64], *SHOC2* [65], *MAP2K1* (*MEK1*) and *MAP2K2* (*MEK2*) [64], *CBL* [66] and *NF1* [67] have been associated with RASopathies in which CHD are observed (Table 1).

### Genes encoding cardiac transcription factors

Cardiac developmental signals are conveyed to transcriptional circuits that regulate gene expression during normal heart development. At the heart of these transcriptional networks lie a set of core transcription factors many of which are associated with isolated CHD (Table 1). Transcriptional focal points include NKX2-5, GATA4 and TBX5. These transcription factors interact at cardiac promoters in synergistic fashions (see below). Their function and molecular signatures have been thoroughly described and excellently reviewed elsewhere, and will only be briefly mentioned here (see [68] for a recent review).

In humans, disease causing mutations in the homeodomain protein NKX2-5 result in a plethora of CHDs including ASD, VSD, TOF and DORV [69–71] Septal defects and atrio-ventricular conduction defects are commonly seen in patients with a mutated *NKX2*-*5* gene [70]. Disruption of *Tinman*, the homologue of *NKX2*-*5* in *Drosophila melanogaster*, results in a fruit fly devoid of the dorsal vessel, a structure analogous to the human heart [72]. Similarly, *Nkx2-5* functionality is crucial in mice as homozygous mutations cause embryonic lethality due to faulty cardiac looping and insufficient myocardial differentiation during chamber formation [73, 74]. Mouse studies show that *Nkx2*-*5* gene dosage is critically important for properly regulated development of the cardiac conduction system as *Nkx2*-*5* null mice lack the primordium of the AV node and the conduction system of heterozygous mutant embryos only contain half the normal number of cells [75]. Additionally, Pashmforoush et al. [76] generated ventricular-restricted *Nkx2*-*5* knockout mice that display progressive complete heart block and massive trabecular muscle overgrowth.

NKX2-5 ranks high in the cardiac regulatory hierarchy and is expressed in both the first- and second heart field (SHF) [77]. Its expression is closely coordinated through GATA factors, SMAD proteins and by self-autoregulation [78–81]. Proliferation of the SHF and outflow tract (OFT) morphology is regulated by Nkx2-5 feedback repression of BMP2/SMAD1 signaling [82]. It was recently shown that JARID2, which is also implicated in OFT development, is a direct target of NKX2-5 regulation [83]. Furthermore, it has been demonstrated that Nkx2-5 interacts with Gata4 within cardiac promoters, suggesting that the proteins cooperate in the transcriptional activation of cardiac target genes [84, 85]. Nkx2-5 interacts with Tbx5 in vitro and the two proteins were shown to activate a cardiac-specific Nppa promoter in a synergistic fashion [86] and Nkx2-5 cooperates with Tbx5 in development of the cardiac conduction system in vivo [87].

GATA binding protein 4 (GATA4) plays a pivotal role near the top of the transcriptional cascades that control heart development (see [88] for a recent review). In humans, the cardiac defects found in patients with interstitial deletions in 8p23.1 are attributed to haploinsufficiency of *GATA4* (see below). Intragenic *GATA4* mutations can also cause isolated CHDs, primarily cardiac septal defects, but PS, TOF and other defects have been reported [89–91]. In a recent paper human missense mutations in *GATA4* were shown to disrupt GATA4–SMAD4 interactions in the BMP/TGF-β signaling pathway, likely causing AVSD and valve abnormalities in the affected patients [92]. Embryonic development in *Gata4* deficient mice is arrested at E10.5 with incorrect ventral folding, endodermal malfunctions and an inability to establish a primitive heart tube [93, 94]. Correct Gata4 dosage is critically important for normal heart development, as mice homozygous for a hypomorphic allele develop CAVC, DORV and a hypoplastic ventricular myocardium [95]. Furthermore, it has also been shown that mice heterozygous for GATA4 mutations develop septation and endocardial cushion defects [90]. Heterozygous knock-in mice harbouring a *Gata4 G296S* mutation, previously identified in patients with septum defects and pulmonary valve stenosis, display ASD and semilunar valve stenosis [96].

GATA4 interacts with numerous transcription factors that promote cardiogenesis [88]. Direct downstream targets of GATA4 include *HAND2* and *MEF2C* required for SHF development [97, 98]. GATA4 and TBX5 proteins physically interact and this interaction is disrupted by mutations in *GATA4* [89]. Furthermore, *Gata4* and *Tbx5* double heterozygous mice develop cardiovascular defects, which point towards a genetic interaction between the two [99].

The T-box transcription factors are important cardiac transcription factors. They are involved in fundamental cardiac developmental processes, including development of the chamber myocardium, outflow tract and the conduction system [100]. TBX1 regulates proliferation of cardiac progenitors in the SHF and haploinsufficiency of *TBX1* is considered the primary cause of CHD in patients with DiGeorge syndrome (see below). TBX5 participates in regulation of gene expression in the developing chamber myocardium and conduction system [101]. Mutations in *TBX5* cause Holt–Oram syndrome (OMIM #142900), a syndrome distinguished by upper limb defects and heart defects—primarily septal and conduction defects [102, 103]. *Tbx5* null mice possess a deformed linear heart tube and underdeveloped atria while heterozygous *Tbx5* mice model heart and limb abnormalities observed in Holt–Oram syndrome, potentially explaining cardiac conduction system defects seen in these patients [101].

Human mutations in *TBX20* cause aberrant valvulogenesis, septal defects, TOF and cardiomyopathy [104]. Deletion of *Tbx20* in mice generates a linear heart tube which fails to loop properly and exhibits insufficient chamber formation [105]. Heterozygous *Tbx20* mice show onset of dilated cardiomyopathy recapitulating some of the human defects [106]. Recent papers by Cai and co-workers [107, 108] place TXB20 in the formation of the cardiac atrioventricular canal in a complex signaling network involving TBX20, TBX2 and BMP2.

In vitro, transcription factor AP-2gamma (TFAP2C) has been shown to bind the *TBX20* promoter site and repress *TBX20* expression [109]. Interestingly, mutations in *TFAP2B* causes Char syndrome (OMIM #169100) characterized by facial dysmorphism, anomalies of the fifth finger and patent ductus arteriosus (PDA) [110]. Isolated PDA has also been linked to mutations in *TFAP2B* [111, 112] and a recent *Tfap2B* knock out study in mice reported phenotypes resembling the characteristics of Char syndrome [113]. TFAP2 isoforms form a complex with CITED2, CREBBP and EP300 [114, 115]. Mutations in the transcriptional co-activators CREBBP and EP300 are associated with Rubinstein–Taybi syndrome (OMIM #180849) displaying mental retardation, broad thumbs and toes, facial abnormalities, and in some cases, CHD [116, 117].

### Genes encoding components of the cardiac sarcomere

Mutations in genes encoding cardiac structural proteins have also been connected to CHD. Several studies link mutations in the cardiac sarcomeric protein MYH6 (myosin heavy chain 6) to ASD [118–120]. Morpholino knock down of *myh6* in the developing chicken heart implies that its functionality is required in the formation of the atrial septum [120]. Molecular regulation of MYH6 expression involves transcription factors such as GATA4 [121], TBX5 and MEF2C [122].

Other members of the contractile units in cardiovascular muscle include MYH7 and ACTC1. A mutation in *MYH7* encoding myosin heavy chain 7 was shown to cause CHDs such as Ebstein’s anomaly and septal defects [123]. Mutations in *ACTC1* encoding the human α-cardiac muscle actin can cause ASD and morpholino knock down of *Actc1* causes looping and atrial septal anomalies in chicken embryos [124].

Mutations in *MYH11*, encoding the major contractile protein of smooth muscle cells can cause thoracic aortic aneurysm and/or aortic dissection and PDA [125]. Mice homozygous for deletion of *Myh11* show a delayed closure of the ductus arteriosus [126], which is connected to the shunting functions of smooth muscle cells upon birth [127, 128].

### Genes encoding chromatin modifiers

Analysis of model organisms has shown that dynamic modification of chromatin structure serves as an important regulator of gene expression during heart development (reviewed in [129]). Genes that encode proteins which modify or bind to histones have been implicated as disease genes in syndromes causing heart defects. This evidence supports a functional link between chromatin modification and human heart development and defects.

Kabuki syndrome (OMIM #147920) is characterized by intellectual disability, craniofacial anomalies, skeletal and hand malformations. Abnormal organ development is also recurrent and includes CHD in approximately 50 % of the cases [130]. Heart defects usually present in the form of septal defects and CoA [131]. Recently, Ng et al. [132] used exome sequencing to identify mutation of *KMT2D* (*MLL2*) as a major cause of Kabuki syndrome. *KMT2D* encodes a histone methyltransferase involved in di- and tri-methylation of the Lys-4 position of histone H3, which marks actively transcribed genes [133].

CHARGE syndrome (OMIM #214800) is characterized by growth retardation and malformation of eyes, ears, genitals, choanae and heart defects—often in the form of outflow tract malformations [134–136]. Approximately two-thirds of the cases are caused by mutation of *CHD7*, which encodes a member of the chromodomain helicase DNA binding (CHD) family [134, 135, 137]. In vitro studies have shown that CHD7 binds DNA regions which correlate closely to regions of H3K4 methylation and regions with characteristics of enhancer elements. This hints that the protein is involved in transcriptional activation [138]. Recently, it was shown that CHD7 controls core components of the transcriptional circuit of neural crest cells and that CHD7 is essential for neural crest cell migration [139]. This function may explain the high frequency of outflow tract defects in CHARGE syndrome, as neural crest cells are known to play a crucial role in septation of the cardiac outflow tract [140].

In a recent study, Zaidi et al. [141] conducted a comprehensive screening of all protein coding genes in hundreds of patients with severe forms of CHD. In this study, whole-exomes of 362 children with CHD and their healthy parents were screened for de novo nucleotide variants by next-generation sequencing (NGS). De novo variants from these parent-offspring trios were compared to de novo variants identified in 264 healthy parent-offspring trios. The authors performed transcriptome profiling experiments to identify genes with a high expression in mouse embryonic hearts (HHE genes). In the trio datasets, they compared the number of de novo variants in genes, homologous to HHE genes. This comparison yielded a significant higher rate of de novo mutations in CHD patients compared to controls. When they compared the frequencies of damaging mutations (i.e. splice-site mutations, nonsense mutations and mutations introducing frameshift) between the two groups, the differences were even more pronounced, with an odds ratio of 7.5 (*p* = 0.001). Interestingly, GeneOntology analysis of 249 de novo mutations identified in CHD patients revealed significant enrichment for mutations in genes involved in H3K4 methylation. Moreover, in CHD patients 27 % of the damaging mutations within HHE genes were affecting proteins involved in H2K4 or H3K27 histone modification. These data suggest that genes involved in histone-modification are significant in the pathogenesis of isolated CHD.

Human genome analysis in combination with functional analysis of candidate genes in animal models has been instrumental in identifying the genes responsible for heart defects in several microdeletion syndromes (see below). Interestingly, several of these genes also encode chromatin modifying proteins, which support the potentially significant role of epigenetic mechanisms in both isolated and syndromic CHD.

## Part II: chromosomal aberrations in congenital heart disease

Microscopically visible chromosomal aberrations are present in 8–18 % of CHD patients [142–144]. Furthermore, CHD is a characteristic part of the clinical spectrum in a significant number of syndromes caused by a chromosome abnormality. The most common chromosome syndrome associated with CHD is Down syndrome [145]. Congenital heart defects are seen in 45 % of individuals with Down syndrome, with the majority of cardiac defects being AVSD, ASD and VSD [146]. Cardiac defects are also found at a high frequency in other aneuploidy syndromes, including Turner syndrome (monosomy X), Edward syndrome (trisomy 18) and Patau syndrome (trisomy 13) [147–150].

CHD is a component of the clinical spectrum in a number of syndromes caused by submicroscopic chromosomal deletions or duplications (listed in Table 2). Some of these syndromes are well-studied microdeletion syndromes, for which the molecular defect has been known for many years. In addition, several novel microdeletion and microduplication syndromes associated with CHD have recently been discovered due to the widespread use of molecular cytogenetic methods.

**Table 2:** Microdeletion and microduplication syndromes with genomic copy number variation and CHD

Syndrome	Chromosome region	Frequency of CHD among patients (%)	CHD candidate gene(s) in region^a^	Function of candidate gene(s)^e^	Reference (OMIM #)
Microdeletion syndromes					
8p23.1 deletion syndrome	8p23.1	94	***GATA4***	GATA-binding TF	[195]
17q23 microdeletion syndrome	17q23	86	***TBX2***	T-box TF	[216] (613355)
DiGeorge syndrome	22q11.2	65–75	***TBX1***; ***CRKL***	T-box TF; Tyrosine kinase	[263] (188400)
1p36 deletion syndrome	1p36	71	***DVL1***	WNT signaling component	[264] (607872)
2q31.1 microdeletion syndrome	2q31.1	70	***SP3***	Sp TF	[265]
Kleefstra syndrome	9q34	40	*EHMT1*	H3K9 methyl transferase	[205, 206, 208] (610253)
16p12.2–p11.2 microdeletion syndrome	16p12.2–p11.2	60	N/A		[266] (613604)
Jacobsen syndrome	11q23-qter	56	N/A		[267] (147791)
Wolf–Hirschhorn syndrome	4p16.3	50	***WHSC1***; ***FGFRL1***	H3K36 methyl transferase; fibroblast growth factor receptor	[268] (194190)
Williams–Beuren Syndrome	7q11.23	53–85	***ELN***; ***BAZ1B***	Elastin; subunit of chromatin remodeling complex	[180] (194050)
Smith–Magenis syndrome	17p11.2	40–45	***MAPK7*** ^b^	MAP kinase	[269, 270] (182290)
Koolen-De Vries syndrome	17q21.31	27–36	*KANSL1* ^c^	Subunit of NSL histone acetylation complex	[210, 212] (610443)
1q21.1 deletion syndrome	1q21	29	***GJA5***	Connexin 40	[197, 271]
Miller–Dieker lissencephaly syndrome	17p13.3	22	N/A^d^		[272, 273] (247200)
Sotos syndrome	5q35	21	*NSD1*	H3K36 methyl transferase	[274–276] (117550)
Brachydactyly-mental retardation syndrome	2q37	20	*HDAC4*	Histone deacetylase	[277, 278] (600430)
15q13 microdeletion syndrome	15q13	15	N/A		[279, 280] (612001)
Microduplication syndromes					
16p13.3 microduplication	16p13.3	40	*CREBBP*	Histone acetyltransferase	[281] (613458)
16p13.11 microduplication	16p13.11	20	***MYH11***	Smooth muscle myosin HC	[282]
Potocki–Lupski syndrome	17p11.2	50	***MAPK7***	MAP kinase	[283, 284] (610883)
22q11.2 duplication syndrome	22q11.2	15	***TBX1***	T-box TF	[285] (608363)
Chromosomal aneuploidy					
Patau syndrome	47, +13 (trisomy 13)	86	N/A		[150]
Edward syndrome	47, +18 (trisomy 18)	61–94	N/A		[147, 148]
Down syndrome	47, +21 (trisomy 21)	50	N/A		[146] (190685)

^a^Genes causing heart defects when deleted in mice (http://www.informatics.jax.org) (bold) and/or by point mutations in additional patients with CHD as part of the clinical spectrum (underlined)

^b^Point mutations in *RAI1* causes Smith–Magenis syndrome, but CHD have not been reported in patients with point mutations in *RAI1* and cardiac defects are not observed in the *Rai1* mouse model [286]

^c^One out of four KDVS patients with point mutations in *KANSL1* had CHD

^d^The lissencephaly phenotype of MDLS is caused by haploinsufficiency of the *PAFAH1B1* gene (also known as *LIS1*) [287, 288], but it is presently unknown which gene is responsible for heart defects in MDLS patients

^e^ *TF* transcription factor

### CHD candidate genes identified from microdeletion and microduplication syndromes

Genotype–phenotype comparisons in patients with microdeletion and microduplication syndromes have identified candidate CHD disease genes. Subsequent mutation screening of candidate genes in patients and studies of the genes and their product in animal models have substantially added to the understanding of CHD and cardiac developmental biology.

The majority of DiGeorge syndrome (DGS, OMIM #188400, also known as 22q11.2 deletion syndrome and velocardiofacial syndrome) are caused by a 3 Mb deletion in 22q11.2 [151]. 22q11.2 duplication syndrome (OMIM #608363) is caused by duplication of genomic material in 22q11.2. Most of the 22q11.2 duplications that have been reported are reciprocal to the common 3 Mb deletion involved in DGS [152].

The common 3 Mb deletion affects more than 50 genes, including the gene encoding the T-box transcription factor TBX1. It is generally accepted that haploinsufficiency of *TBX1* significantly contributes to the CHD phenotype in DGS patients. *Tbx1* ^−*/*−^ mice display similar cardiac phenotypes to individuals with 22q11.2 DS [153, 154]. Conditional knock-out experiments in mice have shown that Tbx1 is required for proliferation of cardiac progenitors in the SHF—a cell population which contributes to the development of the cardiac outflow tract [155]. Additionally, point mutations in *TBX1* have been reported in patients without the 22q11.2 deletion, but exhibit a clinical presentation similar to DGS [156, 157].

Transgenic mice overexpressing *Tbx1* display phenotypic similarities consistent with 22q11 duplication patients, including cardiac outflow tract defects. This suggests that correct gene-dosage of *TBX1* is important for normal cardiac development [152, 158, 159].

Conversely, cases with cardiac defects carrying smaller (1.5 Mb) deletions within the common 3 Mb region, distal to *TBX1* have also been reported [160–162], which implies that other genes in the 3 Mb region may contribute to the cardiac phenotype of DGS patients. An interesting candidate gene within this region is *CRKL*, encoding a protein kinase. Mice with targeted deletion of *Crkl* exhibit defective OFT development and VSDs [160, 163]. Furthermore, experiments with compound heterozygous *Tbx1* ^+*/*−^, *Crkl* ^+*/*−^ mice indicate a possible genetic interaction between the two genes, leading to the increased severity of the cardiac phenotypes in the double mutants [164]. A genetic interaction between *Crkl* and *Fgf8* has also been shown [165], supporting a link among FGF8, TBX1 and CRKL in the pathogenesis of DGS (see below).

A high degree of phenotypic variability is a characteristic feature of DGS and 22q11.2 duplication syndrome. Parts of this variation may perhaps be explained by variations in genes located within 22q11.2, with *TBX1* as the most likely candidate. An alternative explanation involves epistasis, as TBX1 has been shown to regulate or interact with several proteins and signaling networks. Gene expression profiling of tissues in the pharyngeal region from mouse models with targeted deletion of *Tbx1*, have identified several Tbx1 target genes, which include genes involved in homeostasis of retinoic acid (RA) [166–168]. Interestingly, RA regulates *Tbx1* expression [169], thus there seems to be a dual relationship between TBX1 expression and RA signaling. During development of the pharyngeal arches TBX1 expression in the pharyngeal endoderm is regulated by the Hedgehog signaling pathway though the action of forkhead transcription factors [170, 171]. TBX1 itself regulates the expression of FGF8 in the SHF and in the pharyngeal endoderm [172, 173]. A genetic interaction among *Tbx1*, *Six1/Eya1* and *Fgf8* was recently demonstrated in mouse models [174]. Further, TBX1 can act as a negative modulator of BMP signaling by binding SMAD1 and hereby interfere with the SMAD1/SMAD4 interaction [175].

Williams–Beuren syndrome (WBS, OMIM #194050) is caused by deletion of genomic material in 7q11.23. Most patients with WBS are heterozygous for a 1.5–1.8 Mb deletion encompassing ~28 genes [176–179]. Cardiovascular abnormalities are present in 75 % of individuals with WBS, predominantly in the form of supravalvular aortic stenosis (SVAS) and pulmonary arterial stenosis [180]. In 6–10 % of cases aortic or mitral valve defects are also seen, and other so-called “atypical” cardiac defects in the form of ASD, VSD and TOF are observed in a significant fraction of the patients [180, 181].

The *ELN* gene, encoding elastin, is believed to be the gene responsible for SVAS in WBS. Patients with atypical deletions including only *ELN* and *LIMK1* genes and SVAS have been reported [182, 183]. In addition, point mutations in *ELN* are associated with familial and sporadic SVAS [184, 185]. Targeted deletion of the *Eln* gene in mice results in reduced aortic lumen diameter due to subendothelial accumulation of smooth muscle cells [186].

However, deletion of *ELN* does not explain the occurrence of the atypical heart defects in a proportion of WBS patients. Results gained from a recently reported mouse model with targeted deletion of *Baz1b*, indicate that deletion of this gene may account for these defects. *BAZ1B* is located within the WBS common deleted region, and homozygous *Baz1b* ^−*/*−^ mice exhibit a range of cardiovascular defects, which include ASD, VSD, trabeculation defects, coarctation of the aorta (COA), hypoplastic pharyngeal arch artery and a low frequency of DORV [187]. *BAZ1B* (also known as *WSTF*) acts as a subunit in three ATP-dependent chromatin remodeling complexes; the WSTF including nucleosome assembly complex (WINAC) [188], the WICH complex (WSTF-ISWI chromatin remodeling complex) [189] and the B-WICH complex [190]. These complexes are important for gene regulation, DNA replication and DNA repair [189, 191]. Thus, the cardiac phenotypes of *Baz1b* knockout mice and the chromatin remodeling function of BAZ1B suggests that some of the phenotypes involved in WBS, including “atypical” heart defects, may be caused by epigenetic effects.

Wolf–Hirschhorn syndrome (WHS, OMIM #194190) is caused by microdeletions in 4p16.3. Genotype–phenotype comparisons in patients with submicroscopic deletions suggest that haploinsufficiency of the gene encoding the histone lysine methyl transferase WHSC1 (also known as NSD2) contributes significantly to the WHS phenotype [152, 192]. A recently published investigation of mice with targeted deletion of the H3K36me3-specific histone methyltransferase gene *Whsc1* puts forth *Whsc1*as another component in heart development [193]. The *Whsc1* ^−*/*−^ mutant mice displayed ASD and VSD, and co-immunoprecipitation experiments with nuclear extracts prepared from embryonic hearts showed that Whsc1 interacts with the cardiac transcription factor Nkx2-5. Furthermore, ChIP assays demonstrated that Whsc1 cooperates with Nkx2-5 in the transcriptional regulation of target genes. Cross-breeding experiments with *Whsc1* ^−*/*+^ and *Nkx2*-*5* ^−*/*+^ mice suggested a genetic interaction between the two genes during cardiac septal formation. Another candidate gene for heart defects in WHS is the *FGFRL1* gene, which encode a member of the fibroblast growth factor receptor family. During mouse development *Fgfrl1* is expressed in the brain, cranial placodes, pharyngeal arches, somites and heart [194]. Targeted deletion of *Fgfrl1* in mice can result in a range of developmental defects, including heart defects in the form of VSD, and both semilunar and atrioventricular valve deformation [194].

Cardiac defects are observed in 94 % of cases with interstitial deletions in 8p23.1 [195]. The defects range from isolated septal defects to complex heart defects like TOF and hypoplastic left heart syndrome (HLHS). A proportion of the patients carry a ~3.7 Mb recurrent deletion flanked by low copy repeats, although some patients have larger deletions that may extend to the 8p telomere. The gene encoding the cardiac zinc finger transcription factor GATA4 is located within the recurrent deletion, and it is well documented that this gene is associated with congenital heart defects. Mutations in *GATA4* cause human CHD, often in the form of septal defects, but other defects have been reported [89, 91, 196] Mice homozygous for targeted deletion of *Gata4* display early defects in cardiogenesis [93, 94], and phenotypic characterization of mice homozygous for a hypomorphic allele of *Gata4* supports that haploinsufficiency of GATA4 can cause CHD [95]. GATA4 interacts with several other transcription factors during cardiac development, including NKX2-5, TBX5, ZFPM2 (FOG2), SMAD4 and HAND2 [34, 89, 92, 121] (see above). Therefore it is possible that the complex cardiac phenotypes observed in a subset of 8p23 deletion patients are evoked by epistatic effects from genes encoding GATA4 binding partners.

### Other microdeletion and microduplication syndromes which comprise CHD

The widespread use of molecular cytogenetic methods like fluorescent in situ hybridization (FISH) and especially array comparative genome hybridization (array CGH) in clinical genetics laboratories has led to the recent delineation of a number of microdeletion and microduplication syndromes, which incorporate CHD as a component of their clinical spectrum (Table 2).

The minimal deleted region in 1q21.1 deletion syndrome contains the *GJA5* gene [197]. A recent screen of 807 TOF cases revealed significant enrichment of small duplications encompassing *GJA5*, thus providing convincing evidence for a link between GJA5 and CHD [152, 192, 198]. Cardiac defects have been reported in a proportion of mice with targeted deletion of *Gja5* [199, 200], suggesting that haploinsufficiency of *GJA5* may be responsible for cardiac defects in some individuals with 1q21.1 deletions. *GJA5* encodes the cardiac gap junction subunit Connexin 40, which is expressed in the atrial myocardium and the atrioventricular conduction system [201, 202]. Gap junctions are cell membrane channels that interconnect the cytoplasm of neighboring cells. In the heart, these channels contribute to the atrioventricular conduction [203, 204], but at present there is no proposed mechanism describing how *GJA5* haploinsufficiency results in structural heart defects.

Molecular delineation of 9q34 microdeletions and mapping of the chromosomal breakpoints in a patient with a t(X;9) translocation suggested that the *EHMT1* gene is responsible for Kleefstra syndrome (KS, OMIM #610253) [205–207]. Mutation screening in patients without deletions in 9q34 subsequently confirmed that haploinsufficiency of *EHMT1* causes KS [205, 208]. Approximately 40 % of patients with KS and deletion of 9q34 have CHD, and the presence of CHD in five out of eleven KS patients with point mutations in *EHMT1* confirm that this gene is responsible for CHD in KS. *EHMT1* encodes euchromatic histone-lysine N-methyltransferase 1, which regulates transcription by methylation of histone H3 lysine 9 (H3K9Me2) in euchromatic DNA [209].

Koolen-De Vries syndrome (KDVS, OMIM #610443) is caused by recurrent deletions in 17q21.31. Between 27 and 36 % of KDVS patients have CHD [210, 211]. Recent delineation of the critical region of 17q21.31 and mutation screening of KDVS patients without deletion of 17q21 revealed that KDVS is caused by haploinsufficiency of *KANSL1* [212, 213]. One out of four patients with point mutations in KDVS has CHD, hinting that *KANSL1* is a CHD disease gene, although further patient data is needed to confirm this link. *KANSL1* encodes a member of the male specific lethal (MSL) complex initially described in *Drosophila* (reviewed in [214]). Within the MSL complex KANSL1 interacts with KAT8, a histone acetyltransferase which regulates gene expression through acetylation of H4 lysine 16 (H4K16) [215].

Another interesting CHD candidate gene is *TBX2*, which is located within the deleted region in 17q23 deletion syndrome [216]. *TBX2* is expressed in non-chamber myocardium of the developing heart, and mice with targeted mutation in *Tbx2* have defects in the development of the atrioventricular canal (AVC) and the OFT [217]. It has been hypothesized that TBX2 is involved in cardiac chamber development and functions as a local repressor of the chamber-specific gene program in non-chamber regions like the AVC and OFT [218, 219].

### Pathogenic copy number variants identified in cohorts of CHD patients

Array CGH and similar methods have been used to screen cohorts of CHD patients for pathogenic CNVs in the form of duplications and deletions. Since 2007, 14 whole-genome CNV screening studies have been reported, comprising more than 5,000 patients (Table 3, [220–234]). The reported studies show large differences, which include size and phenotypic composition of patient cohorts and the experimental and analytical setup, thus it is somewhat difficult to compare the results. Nevertheless, we find it safe to conclude that pathogenic CNVs are found among a significant portion of CHD patients.

**Table 3:** CNV screens in patients with heart defects

Patients	Phenotype of patients^d^	Microarray type	Main results	Candidate genes in CNVs^g^	Reference
60	Congenital heart disease and extracardiac abnormalities	In-house-made microarray containing BAC/PAC^e^ clones. Average genomic distance of probes were 1 Mbp	CNVs considered to be causal were identified in 10 (17 %) patients	*EHMT1*, ***NKX2-5***, ***NOTCH1***, ***NSD1***	[233]
105	Congenital heart disease with and without extracardiac abnormalities. Subjects with documented syndromes were excluded	In-house-made microarray containing 32 k overlapping BAC clones	Rare de novo or inherited CNVs (0.34–13.9 Mb in size) were detected in 18 (17 %) patients	***GJA5***, ***LTBP1***, ***TBX1***	[222]
40	Congenital heart disease with and without extracardiac abnormalities	NimbleGen Systems, Inc. whole-genome 385 K oligo array	Seven large CNVs were identified in 5 (12.5 %) patients	*N/A*	[230]
114	Tetralogy of Fallot (TOF)	Affymetrix Genome-Wide Human SNP Array 6.0	Eleven (9.6 %) rare de novo CNVs (>20 kb) were identified in 114 TOF trios	***JAG1***, ***NOTCH1***, *RAB10*, ***RAF1***, ***TBX1***	[225]
150^a^	Congenital heart disease and extracardiac abnormalities	In-house-made microarray containing BAC/PAC clones. Average genomic distance of probes were 1 Mbp	CNVs considered to be causal were identified in 26 (17.3 %) patients	*ATRX*, ***CREBBP***, *EHMT1*, *FOXC1*, ***GATA4***, ***NOTCH1***, *RAI*, ***TBX1***	[220]
46	Isolated congenital heart disease	Affymetrix Genome-Wide Human SNP Array 6.0	De novo CNVs were identified in two (4 %) patients	***GJA5***, ***NOTCH1***, ***PDGFRA***, ***TBX1***	[221]
58	Congenital heart disease and extracardiac abnormalities	Affymetrix GeneChip 100 K microarray	Potentially pathogenic CNVs (0.2–9.6 Mb in size) were detected in 12 (20.7 %) patients	***ADAM19***, ***HAND1***, ***MESP1***, ***NRP1***, ***NTRK***	[224]
53	Hypoplastic left heart syndrome (HLHS)	Agilent customized genome-wide 400 K array	Thirty-three rare non-polymorphic CNVs (2–1,554 kb in size) were detected in 25 (47 %) patients	***BMPR2***, ***ZNF423***	[227]
262	Heterotaxy (patients with D-transposition of the great arteries were also included in the sample)	Illumina 610Quad Beadchip platform	Forty-five previously unrecorded genic CNVs (0.27–25 Mb in size) were identified in 39 (14.5 %) patients. A significant (*p* = 1.5e − 4) burden of rare genic CNVs were found in HTX cases (14.5 %) compared to controls (7.4 %)	***GALNT11***, ***NEK2***, ***NUP188***, ***ROCK2***, ***TGFBR2***	[223]
43	HLHS	NimbleGen Systems, Inc. whole-genome 385 K oligo array	A significant (*p* < 0.03) burden of CNVs were found in patients (4.6/subject) compared to controls (2.94/subject). The burden of unique CNVs in CHD patients was not found to be significant	N/A	[229]
67^b^	Left-sided congenital heart disease (BAV, AS, COA, HLHS)	Affymetrix Human Genome-Wide SNP Array 6.0	A total of 73 unique inherited or de novo CNVs (>20 kb) were identified in 54 individuals	*ADORA2B*, *ANG*, *CACNA1C*, *COPS3*, *CRMP1*, *CTHRC1*, *ERCC5*, *EVC2*, *FLII*, *GRPEL1*, *HSD17B10*, *ITGA10*, ***LIMS1***, ***MAPK7***, *MFAP4*, *MSX1*, *MTHFD2*, *NCOR1*, *NGEF*, *PLA2G12A*, *PRPSAP2*, *RASD1*, *SBEBF1*, *SMC1A*, *ULK2*	[226]
2,539	Isolated congenital heart disease (808 TOF and 1,448 other CHDs). Subjects with documented syndromes known to cause CHD were excluded	Illumina 660 W-Quad SNP platform	A significant (*p* = 0.008) burden of rare genic CNVs were found in CHD cases (7.8 %) compared to controls (4.4 %)	*CNOT6*, *EDIL3*, ***GATA4***, ***GJA5***, ***HAND2***, ***PPM1K*** *and 13 genes in the WNT-signaling pathway* (*CDH18*, ***CDH2***, *CTBP1*, ***CTNNB1***, *FAT1*, *LRP5L*, ***NFATC1***, *PCDH15*, *PCDHB7*, *PCDHB8*, *PRKCB*, *PRKCQ*, *WNT7B*)	[232]
203 + 511^c^	Congenital heart disease and extracardiac abnormalities.	Customized 105 k oligonucleotide arrays manufactured by Agilent. Average resolution of 30 kb, with denser coverage at disease loci	A total of 55 rare CNVs (>50 kb) were identified in patients from the discovery cohort. Sixteen of these CNVs were identified in the second cohort	***PDE1A***, ***NALCN***, ***ANKRD11***, ***SOX7***, ***GATA4***, ***CRK***, ***CAMTA2***, ***CECR1***	[228]
433	Tetralogy of Fallot-pulmonary atresia or pulmonary atresia and ventricular septal defect. Subjects with documented syndromes were excluded	Affymetrix Genome-Wide Human SNP Array 6.0	47 large (>500 kb) rare CNVs were found in 43 (9.9 %) patients	***ANGPT2***, *ARHGEF10*, ***ARHGEF4***, *BARD1*, *BBS9*, *C12oerf66*, ***CASP1***, ***CASP12***, ***CASP4***, ***CASP5***, *CCDC148*, *CDH19*, ***CHL1***, *CHRM3*, *CHST8*, ***CNDP2***, ***CNN2***, ***CRKL***, ***DISP1***, ***DNAH11***, ***EDIL3***, ***FGF10***, ***FOXO3B***, ***FSTL3***, *FSTL4*, ***GJA5***, *GMDS*, ***GNA11***, ***HIRA***, ***HNF1B***, ***HRIP3***, *IDS*, *KCNB2*, *KIAA1609*, ***LBH***, ***MAPK3***, ***NBEA***, ***NFATC1***, ***NXN***, ***PARD6G***, ***PDS5B***, *PLXNA2*, *PPM1K*, ***PPP4C***, ***PTBP1***, ***RAF1***, ***S1PR4***, *SEMA3D*, *SEMA3E*, ***SFPQ***, ***SLC25A46***, *SNX8*, ***SOX4***, ***SPG20***, ***TBX1***, ***TBX6***, ***TNFSF11***, ***VCAN***, ***WDR18***, *WNK3*, ***ZNF347***	[231]
945	Congenital heart disease with and without extracardiac abnormalities	Affymetrix Genome-Wide Human SNP Array 6.0	Known CHD-related chromosomal abnormalities^f^ were identified in 135 (14.3 %) patients. Large, rare CNVs (0.22–32.1 Mb in size) were identified in 35 (3.7 %) patients	*FKBP6*, *ELN*, *GTF2IRD1*, ***GATA4***, ***CRKL***, ***TBX1***, *ATRX*, *GPC3*, *BCOR*, ***ZIC3***, ***FLNA***, *MID1*, *PRKAB2*, *FMO5*, *CHD1L*, *BCL9*, *ACP6*, ***GJA5***, ***HRAS***, ***GATA6***, *RUNX1*	[234]

Genes known to cause CHD in humans are underlined, genes with reported cardiovascular system involvement (e.g., from targeted deletion in mice) are bold

^a^Includes 60 patients from Thienpont et al. [233]

^b^A total of 174 patients from 67 families

^c^A discovery cohort of 203 patients and a second independent cohort of 511 patients were analyzed

^d^ *AS* aortic stenosis, *BAV* bicuspid aortic valve, *COA* coarctation of the aorta, *HLHS* hypoplastic left heart syndrome, *TOF* tetralogy of Fallot

^e^ *BAC* bacterial artificial chromosome, *PAC* P1-derived artificial chromosome

^f^Trisomy 21 (*n* = 80), trisomy 18 (*n* = 1), 22qDS (*n* = 42), Turner syndrome (*n* = 8), William’s syndrome (*n* = 3), and Triple X syndrome (*n* = 1)

^g^Candidate genes suggested by the authors

The highest frequency of pathogenic CNVs is found among patients with CHD and extra-cardiac anomalies. Based on the current reports [220, 224, 228, 230, 233, 234] we estimate that pathogenic CNVs are present in 15–20 % of patients with CHD and extra-cardiac anomalies.

Among patients with isolated CHD, the frequency of pathogenic CNVs is significantly lower. Here, we estimate the frequency to be between 4 and 14 % [221, 222, 225, 231, 232]. However, this estimate should be treated with caution, due to the aforementioned large differences in study design.

### Identification of disease genes and pathways from CNVs detected in cohorts of CHD patients

In principle, CNVs identified as pathogenic in CHD patients should contain one or more dosage sensitive cardiac developmental genes. Thus, each pathogenic CNV, or at least overlapping CNVs should define a disease locus for CHD and it therefore should be possible to use CNVs to detect CHD disease genes. Several groups have reported identification of CNVs spanning genes, which span genes previously recognized to cause CHD in animal models (Table 3), thereby providing a plausible link between these genes and CHD in humans.

However, many of the identified CNVs do not contain a well-established cardiac developmental gene. These CNVs often contain several genes, although only one is likely to be the gene responsible for CHD. Three approaches have been utilized to identify the causal genes in such cases: (1) narrowing of the locus by comparison of multiple samples with overlapping CNVs, (2) in silico gene prioritization and (3) functional investigations of candidate genes within the CNVs.

Hitz et al. [226] used Endeavour [235] to test for enrichment of angiogenesis-associated genes within 73 CNVs identified in patients with left-sided CHD. They also searched for genes with expression in the developing heart in serial analysis of gene expression (SAGE) and public databases. By combining these prioritization methods, they identified 25 CHD candidate genes (Table 3). Soemedi et al. [232] performed genomic region annotation enrichment analysis on rare deletions and duplications identified in 2,256 CHD cases. They found enrichment of 13 genes encoding proteins involved in the WNT signaling pathway (Table 3). Silversides et al. [231] performed a systematic review of genes within rare CNVs identified among 433 cases with TOF and identified 62 CHD candidate genes (Table 3). They also assessed whether genes, in predefined gene-sets derived from GeneOntology (GO) annotations and pathway and protein domain databases, were significantly overrepresented in CNVs detected in TOF cases compared to controls. They found enrichment of gene-sets belonging to five functional clusters: vasculature development, chromosome organization, cell motility, chemotaxis and neuron projection and development. Lalani et al. [228] identified eight candidate genes in CNVs identified in patients with CHD and extracardiac anomalies (Table 3). They grouped genes within enriched CNVs based on GO categories and found enrichment for genes encoding proteins involved in G-protein coupled receptor internalization, hemopoiesis and cytoskeleton organization. Furthermore they analyzed protein–protein interactions between proteins encoded by candidate genes in CNVs identified in patients and a set of 276 proteins from GO cardiac development categories. They identified 11 proteins with at least one connection with a human cardiac-specific protein (significant at *p* = 0.03).

Thienpont et al. [236] identified *TAB2* as a dosage sensitive CHD disease gene by comparing overlapping CNVs within 6q25. The overlapping region of seven CNVs identified in CHD patients revealed a CHD locus containing 11 candidate genes, including *TAB2*. For prioritization of the candidate genes in the locus and surrounding genomic region, the authors performed in silico analyses of 105 genes in 6q24–25, using an adapted version of Endeavour [235]. This analysis predicted *TAB2* as the highest-ranking candidate gene in 6q24–25. Further functional analyses of TAB2 in human embryonic heart tissues and zebrafish suggested that *TAB2* is a cardiac developmental gene. Point mutations localized within *TAB2* in two unrelated CHD patients and mapping of a translocation breakpoints within *TAB2* in a CHD family segregating a t(2;6) translocation, further verified *TAB2* as a CHD disease gene.

Fakhro et al. [223] performed whole-genome CNV screening of 262 patients with Heterotaxy and isolated TGA. They identified 45 unrecorded gene-containing CNVs, including two different CNVs affecting *TGFBR2*. Evaluation of candidate genes using in situ hybridization and Morpholino-based gene knock-down in *X. tropicalis* showed that the genes *tgfbr2*, *rock2*, *galnt11*, *nek2* and *nup188* are involved in left–right patterning of the heart (Table 3). ROCK2 and NEK2 are ciliary proteins, thus this study confirmed the importance of cilia and TGF-β signaling in LR patterning [237]. In addition, this study identified two novel genes (*GALNT11* and *NUP188*) with unknown functions in LR development.

### The molecular pathology of congenital heart disease

**Fig. 1:**
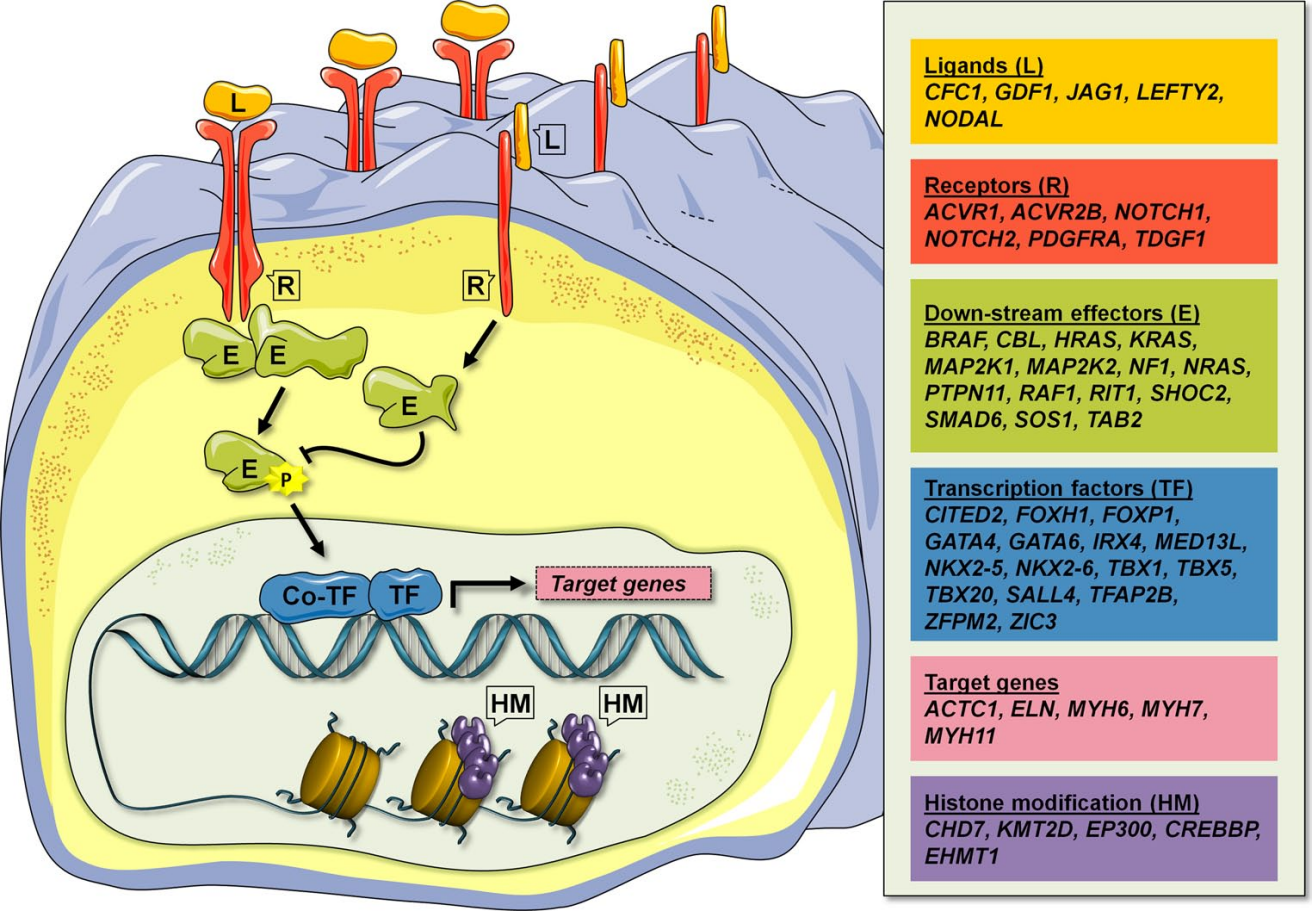
Schematic representation of the different cell signaling components affected by mutations in human CHD disease genes. These include ligands (*L*), receptors (*R*), down-stream effectors (*E*), transcription regulators, which include transcription factors (*TF*), transcription co-factors (*co-TF*) and histone modifying proteins (*HM*), and target genes. Known human CHD disease genes within the six groups are shown in the *panel* at the *right*. *Colored figure* are shown in the on-line version of the article

Cardiac development is controlled by a large number of signaling pathways, which are tightly regulated in time and space, and interact in complex developmental networks [34]. The CHD disease genes, which have been identified to date, suggest that all aspects of developmental signaling pathways may be involved in human CHD: from ligands (e.g. JAG1) and receptors (e.g. NOTCH, PDGFRA), across down-stream signaling effectors (e.g. PTPN11, SMAD6), to transcription factors (e.g. GATA4, NKX2-5) and targets (e.g. ACTC1, MYH6) (Fig. 1). Moreover, discoveries of disease genes encoding histone-modifying proteins (e.g. CHD7, KMT2D), suggest that epigenetic regulation of an unknown number of target genes, may add an additional layer of regulation on consensus cardiac developmental networks.

Lage et al. [238] have recently shown that a wide range of CHD risk factors, functionally converge in complex, yet discrete, protein networks driving heart development. These findings, combined with the potentially hundreds of CHD disease genes [141], suggest that CHD may be caused by a very large number of combinations of mutations and environmental risk factors.

Reduced penetrance of CHD are often observed in human pedigrees (e.g. [239], unpublished observations in Danish pedigrees) and in carriers of CNVs known to cause CHD (see Table 2 and discussions in text). Some of the reduced penetrance may simply be due to unidentified asymptomatic heart defects in some carriers, but may also be caused by epistasis. Several examples of epistasis in mouse models are mentioned in the text above. Winston et al. [240] performed a systematic study of the influence of genetic background on the expression of heart defects in *Nkx2*-*5* ^+*/*−^ heterozygous mice. The authors compared *Nkx2*-*5* ^+*/*−^ heterozygous C57Bl/6 mice with *Nkx2*-*5* ^+*/*−^ heterozygous F1 progeny from crosses with two other mouse strains. The data showed that the F1 hybrid mice presented with a significantly lower incidence of septal defects compared to mice with the original C57Bl/6 background. The authors suggest that modifying alleles can either direct the manifestation of a cardiac developmental defect or buffer the effect from perturbations. The latter situation, which was the case in the study, may ensure robustness of normal heart development.

How the large heterogeneity in CHD and potentially large epistatic effects translates into lesions in the personal genome of the individual patient remains to be investigated. One possible scenario could be that individual combinations of several risk alleles may be the cause of CHD in part of the patients.

### Future perspectives

It was recently demonstrated that exome-sequencing is a powerful tool for identification of de novo mutations in CHD [141]. It is very likely that more studies based on exome-sequencing will reveal new CHD disease genes in the near future. The high number of variants identified in exome-sequencing experiments is a big challenge in very heterogeneous disorders like CHD. Thus, it is likely that such studies will be performed on large numbers of parent-offspring trios or on families with dominant or recessive inherited CHD.

The past 5 years have shown that analysis of genome rearrangements in the form of CNVs, translocations or inversions can lead to detection of new CHD disease genes or loci. The technology for mapping such rearrangements is continuously improving, and breakpoints in balanced translocations and inversions can now be mapped within days using NGS [241].

Untreated, CHD is a disorder with a high mortality rate, therefore a large part of the disease causing mutations are likely rare in populations due to negative selection. However, it is also possible that some variants associated with CHD may escape negative selection. Such variants may be discovered through genome wide association studies, as has recently been demonstrated [242–244].

Interesting therapeutic opportunities could arise from the current knowledge of the molecular pathogenesis of CHD. A significant part of CHD seems to be caused by mutations which perturb complex developmental networks. These networks are characterized by extensive communication within and between specific signaling pathways, and with the environment. Thus, given the apparent epistatic effects observed in patients and animal models, it should be possible to manipulate the signaling pathways in the developmental networks with synthetic agonists or antagonists, and thereby alleviate effects from mutations or redirect signaling events towards normal heart development. A recent study suggests that this could be possible someday. Tian et al. [245] showed that defects in the cardiac inflow tract and AV canal (resembling complete CAVC in humans) in *Wnt2* ^−*/*−^ mice, could be rescued by transient pharmacological activation of Wnt signaling with LiCl.

Naturally, such therapeutic opportunities are presently very hypothetical, and to become reality, much more knowledge about the molecular genetics and the molecular pathology of CHD are needed. Combining human genetics/genomics with functional studies in cell models or animal models like zebrafish, *Xenopus* frogs, chicken or mice are likely to have the greatest impact on our understanding of the molecular pathology in human CHD.
